# Error detection and correction in intracortical brain–machine interfaces controlling two finger groups

**DOI:** 10.1088/1741-2552/acef95

**Published:** 2023-08-25

**Authors:** Dylan M Wallace, Miri Benyamini, Samuel R Nason-Tomaszewski, Joseph T Costello, Luis H Cubillos, Matthew J Mender, Hisham Temmar, Matthew S Willsey, Parag G Patil, Cynthia A Chestek, Miriam Zacksenhouse

**Affiliations:** 1 Department of Robotics, University of Michigan, Ann Arbor, MI, United States of America; 2 BCI for Rehabilitation Lab., Faculty of Mechanical Engineering, Technion - Israel Institute of Technology, Haifa, Israel; 3 Cortical Neural Prosthetics Lab., Department of Biomedical Engineering, University of Michigan, Ann Arbor, MI, United States of America; 4 Department of Electrical and Computer Engineering, University of Michigan, Ann Arbor, MI, United States of America; 5 Department of Neurosurgery, University of Michigan, Ann Arbor, MI, United States of America

**Keywords:** error detection, error correction, intracortical brain–machine interfaces, execution errors, linear filters

## Abstract

*Objective.* While brain–machine interfaces (BMIs) are promising technologies that could provide direct pathways for controlling the external world and thus regaining motor capabilities, their effectiveness is hampered by decoding errors. Previous research has demonstrated the detection and correction of BMI outcome errors, which occur at the end of trials. Here we focus on continuous detection and correction of BMI execution errors, which occur during real-time movements. *Approach.* Two adult male rhesus macaques were implanted with Utah arrays in the motor cortex. The monkeys performed single or two-finger group BMI tasks where a Kalman filter decoded binned spiking-band power into intended finger kinematics. Neural activity was analyzed to determine how it depends not only on the kinematics of the fingers, but also on the distance of each finger-group to its target. We developed a method to detect erroneous movements, i.e. consistent movements away from the target, from the same neural activity used by the Kalman filter. Detected errors were corrected by a simple stopping strategy, and the effect on performance was evaluated. *Main* *results.* First we show that including distance to target explains significantly more variance of the recorded neural activity. Then, for the first time, we demonstrate that neural activity in motor cortex can be used to detect execution errors during BMI controlled movements. Keeping false positive rate below $5\%$, it was possible to achieve mean true positive rate of $28.1\%$ online. Despite requiring 200 ms to detect and react to suspected errors, we were able to achieve a significant improvement in task performance via reduced orbiting time of one finger group. *Significance.* Neural activity recorded in motor cortex for BMI control can be used to detect and correct BMI errors and thus to improve performance. Further improvements may be obtained by enhancing classification and correction strategies.

## 1. Introduction

Spinal cord injury (SCI) leading to quadriplegia or paraplegia affects approximately 300 000 people in the United States [1]. A subset of those affected by SCI are unable to interact with the environment due to dysfunctional hands and arms. Autonomy is significantly impacted, and self-care must be done by a caretaker. Recent improvements in brain recording and in robotic kinematics have provided an opportunity for those with SCI to regain natural body movement. Brain–machine interfaces (BMI) bypass the spinal cord and the non-functioning limb by sampling brain activity to control robotic limb movement or some other output device such as a keyboard [2].

BMIs, whether invasive or not, are prone to prediction errors [3–5]. In typical closed-loop BMI applications, the user receives visual feedback about the action taken by the BMI, so BMI errors would evoke error-processing in the user’s brain [3, 6]. Neural correlates of error processing have been investigated extensively using electroencephalogram (EEG) [7–10], and the resulting potentials are known as error-related potentials (ErrPs). Different ErrPs were associated with outcome errors, i.e. failures to perform the task, and execution errors, i.e. deviations between the executed and expected movement [7, 11]. There is a great interest in detecting ErrPs online to improve EEG-based BMI applications, including automatic correction of the selected character in P300 spellers [12, 13], and automatic undoing and even correction of discrete robotic actions [14, 15].

Error signals have also been detected from intracortical neural activity from premotor and primary motor (M1) cortices to improve invasive BMIs [4]. Using a cursor grid task, Even-Chen *et al* demonstrated that outcome errors can be detected from single trials with high accuracy and that preventing or deleting erroneous selections significantly improved performance. However, that study focused on detecting outcome errors, at the end of a trial, rather than execution errors during a movement. Thus, correction was limited to undoing erroneous selections rather than continuously correcting the movement.

Neural correlates of error-processing have also been investigated using fMRI with humans [16]. Execution errors were introduced by sensorimotor rotations or force fields. In both cases, execution errors activated regions along the central and postcentral sulci. In particular, clusters in the arm area of the contra-lateral M1 were significantly more activated in reaching movements with perturbations than without. In another study, involving invasive BMI experiments with non-human primates, it was demonstrated that the modulations in neural activity recorded from M1 and premotor dorsal (PMd) increase after switching to brain control [17], and that this can be attributed to increasing process noise due to imperfections in the BMI filter [18]. Thus, we hypothesized that the neural activity recorded in the motor cortex includes information about BMI movement errors.

In our earlier work [19] we focused on invasive BMI experiments with non-human primates involving the control of a single finger. Neural activity was recorded with a micro-electrode array implanted in the hand area of precentral gyrus (PCG) to capture motor cortex activity. We demonstrated that it is possible to differentiate between correct and erroneous movements based on the recorded neural activity. However, detection was performed offline and was not used to correct the BMI.

Here we extend our earlier work with invasive BMI experiments to include online error monitoring and correction while two non-human primates controlled either a single or two finger-groups. Our work addressed the following research questions: (1) does the neural activity recorded in motor cortex encode the distance of individual finger groups to the target, and thus can continuous execution errors be seen in patterns of neural activity? (2) How well can erroneous movements be detected based on the neural activity recorded in motor cortex both offline and, most importantly, online? and (3) Is it possible to improve the operation of the BMI by correcting the movements that are detected as erroneous?.

## 2. Methods

### 2.1. Experimental setup

**Figure 1.**
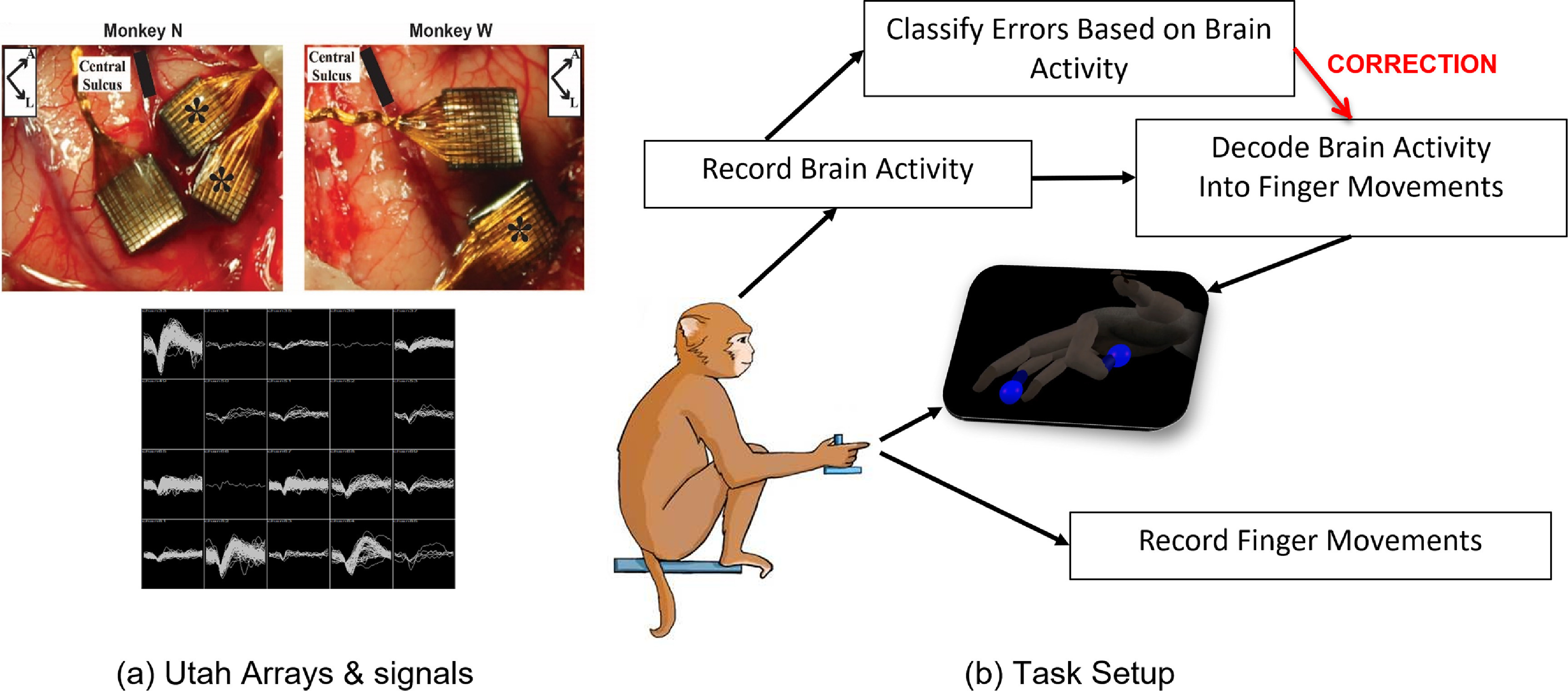
Experimental setup: (a) surgery photos of Utah array implants in both monkeys in the top two panels, and representative spikes from Monkey N’s array in the bottom panel, (b) block diagram demonstrating the flow of information used in the error detection and correction task: neural activity and finger movements are recorded during hand control to train a Kalman filter as the monkey acquires visual targets on a screen; the Kalman filter is then controlled online to train an error classifier; the error classifier is then run in parallel, and detected errors can be corrected by modifying the output of the Kalman filter.

Two adult male rhesus macaques (Monkey W and Monkey N) were implanted with Utah arrays (Blackrock Microsystems, Salt Lake City, Utah) in the hand area of PCG, which typically includes both M1 and PMd in monkey cortex, and one monkey was additionally implanted with a Utah array in sensory cortex (not used in this study), as shown in figure 1(a). The arrays in both monkeys were over one year old at time of study. The monkeys were trained to sit in a chair and use a hand manipulandum to control virtual fingers on a screen and move the virtual fingers to target positions, as illustrated in figure 1(b). The angles of the virtual fingers were determined from bend sensors embedded within the hand manipulandum.

Neural data was recorded from 96 channels within the motor cortex and processed via a Cerebus neural signal processor (Blackrock Microsystems). Spiking-band power (SBP), which was demonstrated to be well correlated with single-unit activity by Nason *et al* [20], was used as the neural feature. The quality of the neural signals was inspected visually, during daily setup, and channels with visible noise were excluded from decoding and analysis. Channels were referenced to the average activity of all the remaining channels as detailed in Ludwig *et al* [21]. During the experimental days analyzed in this study, the number of channels representing an average firing rate over 1 Hz (a baseline metric for spiking activity) ranged from 20 to 47 channels for Monkey N and 11 to 19 channels for Monkey W, as detailed in the last column of table 2. Monkey N was observed to have more tuned neural signals and better task performance than Monkey W in both offline and online control. Differences in decoder performance between monkeys is expected due to the differing brain area targeting, levels of motivation and behavioral performance observed between monkeys [22].

**Figure 2.**
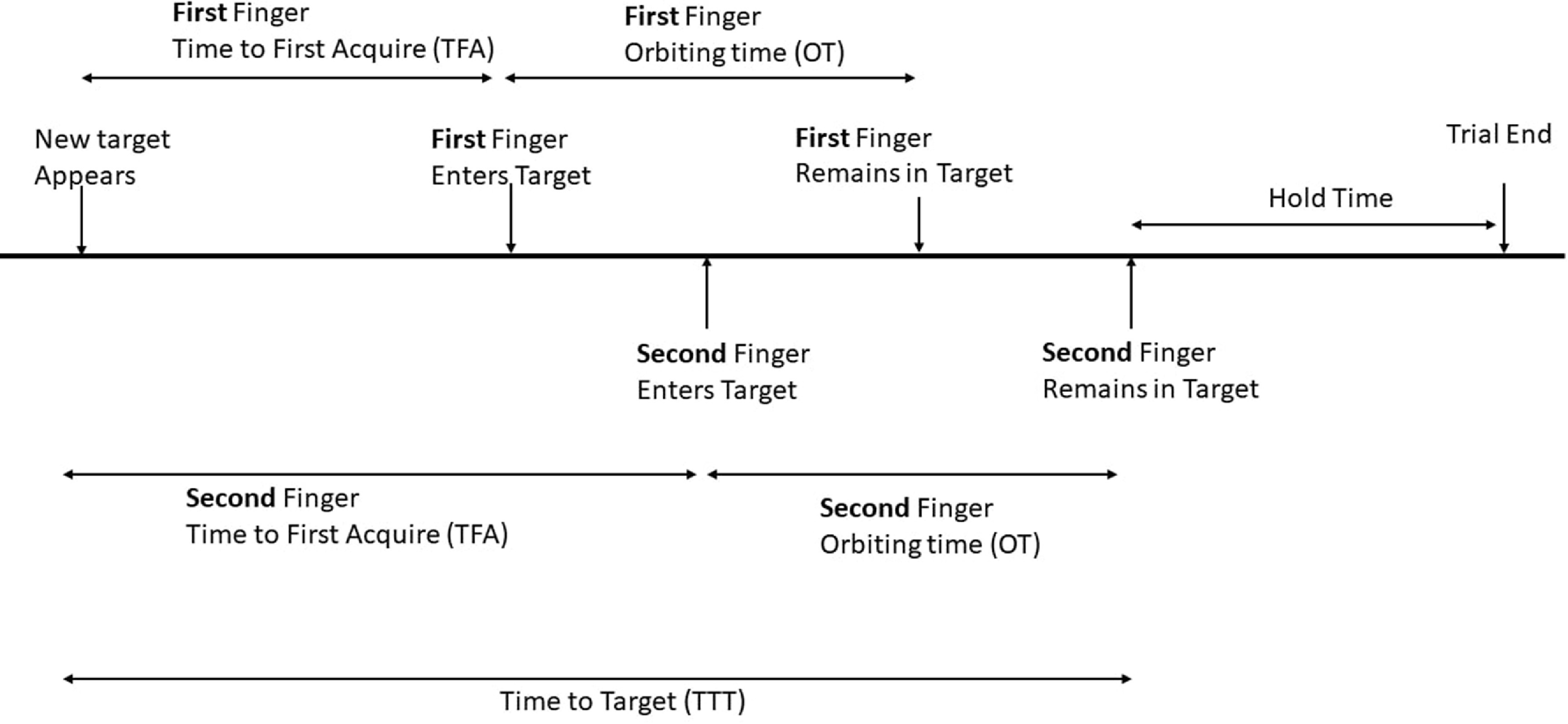
Sequence of events during the two-finger task performed by Monkey N. A trial starts when new targets appear, and the time until a specific finger-group first enters its target is the time to first acquire (TFA). First-finger and Second-finger refer to either the index-finger or MRS finger-group, depending on which finger group first enters its target. Orbiting time (OT) is the duration between TFA and the time after which the finger-group remains in target (until the end of trial), and thus is finger-dependent. For successful completion of the task, both fingers have to remain in the target during the hold time. Total time to target (TTT) is the total trial time minus the hold time. TTT depends on both fingers, and is thus finger-independent. The single-finger task, performed by Monkey W, is similar but involves only one finger, so hold time starts when that finger remains in target till the end of trial.

Monkey W performed a single degree-of-freedom finger task, detailed in Vaskov *et al* [23] while monkey N performed a two-finger task, previously developed in Nason *et al* [22]. Figure 2 outlines the sequence of events during the two-finger task and its simplification in the single-finger task. In the single finger task, the monkey acquired a single target in each trial by moving the index, middle, ring, and small fingers as a single group. For successful completion of the task, the monkey was required to hold the virtual fingers within the target range for a predetermined hold time (750 ms for offline training and 500 ms for online control). For the two-finger task, the monkey used two individual finger-groups to acquire two simultaneous targets along corresponding arcs: the index finger, and the middle-ring-small (MRS) finger-group. The hold time started when both finger groups were in their respective targets, as indicated in figure 2.

### 2.2. Experimental phases

**Figure 3.**
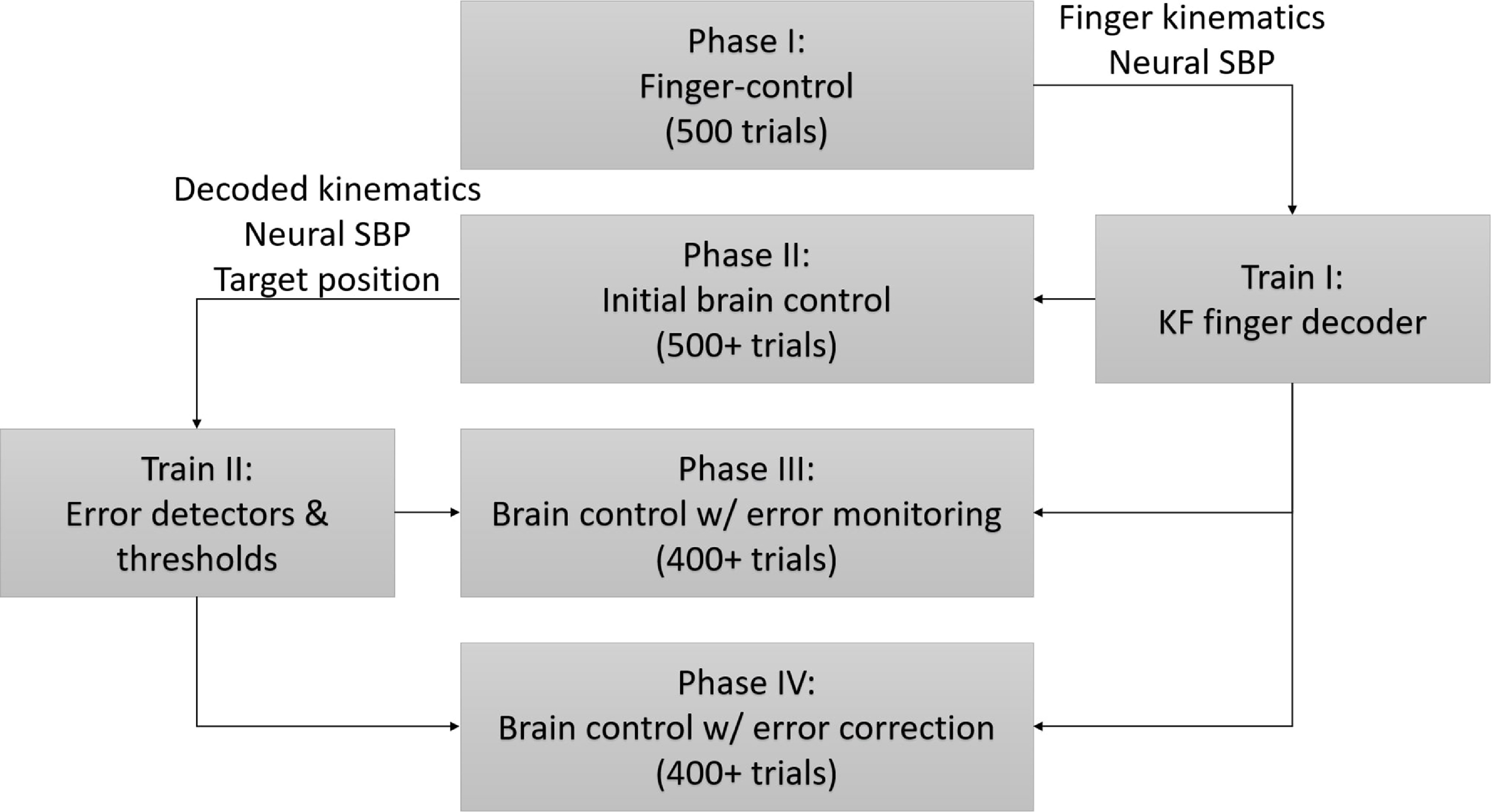
Block diagram of the four phases of the experiment and the two training sessions. Monkey N performed all four phases, while Monkey W performed only the first three phases. Data collected during finger control (phase I, 500 trials) was used to train the Kalman Filter (KF) decoder for finger movement. This decoder was used during all the phases of brain control to decode the neural activity and control the virtual fingers accordingly. Data collected during initial brain control (Phase II, over 500 trials) was used to train the error detectors. The error detectors were evaluated in parallel with the KF decoder, based on the same neural activity, to monitor errors (Phase III, over 400 trials) and to correct detected errors (Phase IV, over 400 trials). Phases III and IV were alternated in an ABA fashion.

In order to enable online error monitoring and correction, we developed two decoders that extract different information from the same neural activity: (1) a velocity decoder that determines the command to the virtual fingers, as detailed in section 2.1, and (2) an error detector that detects activity evoked by erroneous movements, as detailed in section 2.4. The velocity decoder and error detector were trained based on data collected during the first two phases of the experiment, respectively, as illustrated in figure 3. The performance of error monitoring and error correction was evaluated in the last two phases, which were performed only by Monkey N.

The four experimental phases are detailed in figure 3. During Phase I, the monkeys performed 500 trials of the finger task (finger control), while neural data and finger kinematics were simultaneously collected. The recorded data was binned (50 ms) and used to train a velocity-based Kalman filter (KF) decoder, as detailed in [22]. During the following phases (brain control, Phases II, III, and IV), the trained KF was used online to decode the measured neural activity and control the movements of the virtual fingers. During brain control the monkey’s hands were not restricted, but the kinematics of the fingers had no direct effect on the decoder output except for their effect on the recorded neural activity. Data recorded during initial brain control (Phase II, over 500 trials) was used to train error detectors for each finger group and each movement type (flexion or extension) from incoming neural data, and to determine their thresholds, as detailed in section 2.4.

During the next two phases, the trained error detectors were evaluated online in parallel with the KF decoder. During phase III (brain control with error monitoring, over 400 trials), the velocities of the finger-groups were controlled exclusively by the output of the KF decoder. However, during phase IV, the output of the KF decoder was corrected online when erroneous movements were detected (brain control with error correction, over 400 trials), as detailed in section 2.6.

To asses the effect of error correction, another phase of brain control with error monitoring was conducted, without error correction (not shown in figure 3). Thus the last part of the experiment followed an ABA format, where A and B refer to brain control with error monitoring and with error correction, respectively. Experimental days were excluded from analysis if the first run of brain control with error monitoring (A) contained less than 400 trials within the allocated time of 12.5 min. This was done to ensure that the monkey performing the trials was not losing motivation during this run, and thus the comparison between runs is valid.

All experiments were conducted using xPC Target version 2012b (Mathworks) which logged incoming neural activity from the Cerebus processor and finger positions from the bend sensors every 1 ms. Training of both KF decoders and error detectors was performed in MATLAB versions 2012b or 2021b (Mathworks), and parameters were uploaded to files located on a central storage server. In online experiments a computer running Python 3.7, named the eXternal Graphic Processing Computer (xGPC), received binned neural data every 50 ms from the xPC Target computer via network interface, and the KF decoders and error detectors were run in real-time using this data. Finger positions and error classifications were sent back to xPC Target from the xGPC every 50 ms via network interface to update the virtual fingers, using the software architecture detailed in [24]. Real-time kinematics, classification output (when available), and target data were collected for offline analysis.

### 2.3. Variance analysis

It is commonly assumed that the neural activity can be related to the kinematics of arm movements, including position, velocity and speed, via a linear model [25–29] or generalized linear model [30, 31]. It has recently been demonstrated that SBP is related to the kinematics of the finger groups, including the position of each finger-group (i.e. distance along the corresponding arc), *P*, and its rate of change, *V* [22]. Here, we assess the hypothesis that the neural activity also encodes the distance to the target. Specifically, we assess the hypothesis that the distance $D = T-P$, between the position of a finger-group, *P*, and its target, *T*, contributes significantly to estimating the SBP. First we consider a single lag model, which relates the neural activity in bin *k* to the kinematics at a single lag *l*, i.e. to the kinematics at a single-bin *k* + *l*

\begin{align*} \hat{SBP}(k) &amp; = g \large[ \omega_{P_\textrm {I}}(l)P_{I}(k+l)+\omega_{P_\textrm {MRS}}(l)P_\textrm {MRS}(k+l)\nonumber\\ &amp; \quad + \omega_{V_\textrm {I}}(l)V_\textrm {I}(k+l)+\omega_{V_\textrm {MRS}}(l)V_\textrm {MRS}(k+l)\nonumber\\ &amp; \quad + \omega_{D_\textrm {I}}(l)D_\textrm {I}(k+l)+ \omega_{D_\textrm {MRS}}(l)D_\textrm {MRS}(k+l)\nonumber\\ &amp; \quad +\omega_{0} \large] + \epsilon(k,l) \end{align*}

where $\hat{SBP}$ is the estimated SBP, $P_{*}$, $V_{*}$ and $D_{*}$ are the position, (scalar) velocity and distance to the target of the index or MRS finger-groups, as indicated by the subscripts $_\textrm {I}$ and $_\textrm {MRS}$, respectively, $\omega_{*}$ are the corresponding regression weights, *ω*_0_ is the bias parameter, *l* is the lag, $\epsilon(k,l)$ is the residual error, and *g* is a linear or an exponential function, for linear or generalized linear models, respectively. Here we evaluate only the linear model.

We also evaluate the multi-lag model, which extends the single-lag model to account for the kinematics in multiple-lags. Considering the linear model, the neural activity in bin *k* is related to the kinematics in $2L+1$ lags around the neural activity:

\begin{align*} \hat{SBP}(k) &amp; = \sum_{l = -L}^{L}\omega_{P_\textrm {I}}(l)P_\textrm {I}(k+l)\nonumber\\ &amp; \quad +\sum_{l = -L}^{L} \omega_{P_\textrm {MRS}}(l)P_\textrm {MRS}(k+l)\nonumber\\ &amp; \quad +\sum_{l = -L}^{L} \omega_{V_\textrm {I}}(l)V_\textrm {I}(k+l)\nonumber\\ &amp; \quad +\sum_{l = -L}^{L} \omega_{V_\textrm {MRS}}(l)v_\textrm {MRS}(k+l)\nonumber\\ &amp; \quad + \sum_{l = -L}^{L} \omega_{D_{I}}(l)D_\textrm {I}(k+l)\nonumber\\ &amp; \quad +\sum_{l = -L}^{L} \omega_{D_\textrm {MRS}}(l)D_\textrm {MRS}(k+l)+\omega_{0} + \epsilon(k). \end{align*}

The coefficient of determination, *R* ^2^, between $\hat{SBP}$, and the actual SBP describes the fraction of the variance in the actual SBP that is captured by the corresponding model. The contribution of the distance to the target is assessed by comparing *R* ^2^ with and without the distance to the target.

### 2.4. Error detection

Erroneous movements are those that move away from the target, while correct movements are those that move toward the target. We hypothesized that the neural activity, and in particular SBP, encodes whether movements are erroneous or not. While erroneous movements may occur during finger control they are much more prevalent during brain control. Thus, we trained the error detectors on data from initial brain control and tested their performance during brain control with error monitoring and during brain control with error correction.

**Figure 4.**
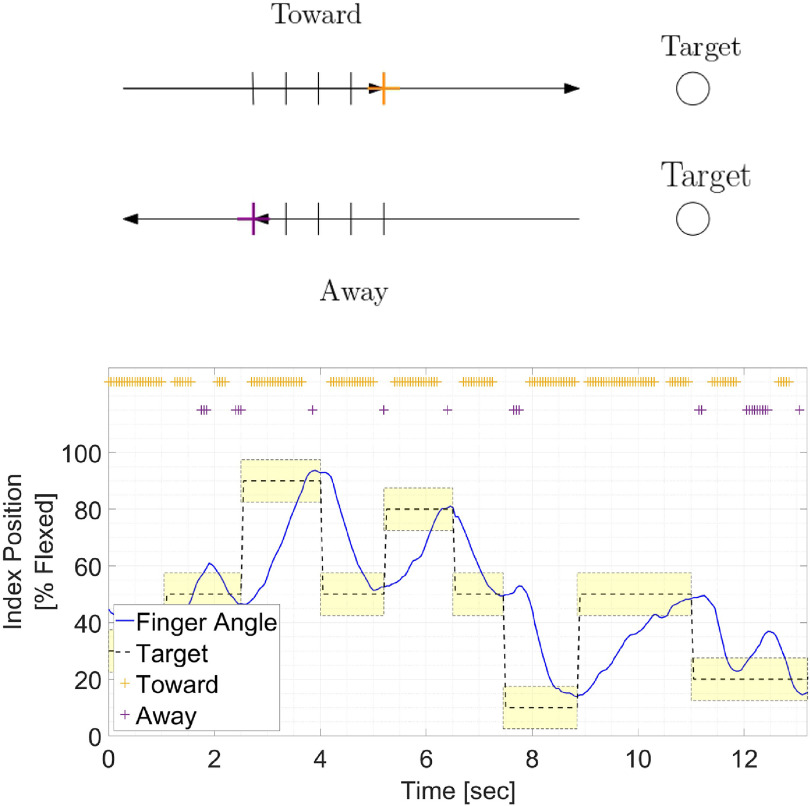
Labeling movement segments as ‘toward’ or ‘away’ from the target. (a) Segments are labeled as ‘toward’ or ‘away’ from the target if the movement in the last *N* bins is consistently toward or away from the target (here *N* = 4). (b) A typical 10 s of decoded finger positions (blue) recorded from Monkey W during initial brain control (day 2) along with target locations (light-yellow) and their centers (dashed black). Toward and away segments (of *N* = 4 bins) are marked by yellow and purple pluses (‘+’), respectively, on top of the graphs.

To avoid confusion and facilitate error detection, we focused on detecting erroneous movements lasting *N* bins. Thus, a moving window of *N* bins was used to generate overlapping movement segments. Segments were labeled as ‘toward’ or ‘away’ if the decoded velocity in each of the *N* bins was toward or away from the target, as shown in figure 4(a) for *N* = 4, and were unlabeled otherwise. The parameter *N* was selected based on the variance analysis detailed in sub-section 3.1.1, which indicated that the neural activity lags the distance to the target by 4–5 bins.

Figure 4(b) depicts the resulting labels for a typical section of 10 s of initial brain control recorded during an experiment with Monkey W. Decoded finger positions are plotted in blue, target locations in light-yellow and their center in dashed black. The corresponding labels of segments of *N* = 4 bins toward and away from the target are marked by yellow and purple pluses (‘+’), respectively, at the top. It is important to note that even when the monkeys eventually acquired the target and performed the task successfully, the movement may not have been consistently toward the target but may have included segments away from the target. This is apparent in figure 4(b), e.g. just before 8 s when the 3 consecutive purple ‘+’ s mark a segment in which the index finger moved away from the target, and just after 12 s when the 9 consecutive purple ‘+’ s mark a segment in which the index finger moved outside the target.

Labeled segments were separated into three groups depending on the decoded velocity: consistent flexion, consistent extension, or inconsistent movement. Labeled segments in the two consistent movement groups were used to train separate error detectors for each finger group and movement type, while segments with inconsistent movement type were excluded. This resulted in two error-detectors for Monkey W and four error-detectors for Monkey N, one for each movement type of each finger group. The input to the error-detectors was *N* samples of SBP from the channels used by the KF.

Each error detector was trained on a balanced set of labeled training segments (same number of ‘toward’ and ‘away’ segments). The threshold was selected from the receiver operating curve (ROC) estimated from a balanced set of labeled validation segments. The ROC describes the trade-off between true positive rate (TPR, i.e. the rate at which segments away from the target are correctly classified as such) versus false positive rate (FPR, i.e. the rate at which segments toward the target are mistakenly classified as away from the target). Thresholds were selected to limit FPR below $5\%$, or to obtain the minimum positive FPR if it remained above $5\%$ for all the evaluated thresholds. ROCs are also useful for comparing the performance of different classifiers. Better classifiers are characterized by larger TPRs for the same FPRs, and thus by a larger area under the ROC curve (AUC).

We considered three well-known classification methods: (1) step-wise linear discriminate analysis (SWLDA, [19]), (2) quadratic discriminate analysis (QDA), and (3) linear support vector machine (SVM). In order to determine which method is most appropriate, we compared the performance of these methods on data recorded during initial brain control using five-fold cross-validation. For each fold, the error-classifier was trained on 80% of a balanced set of labeled segments and the threshold selected from the ROC computed from the remaining 20%. Table 1 compares the resulting average TPRs and FPRs, on four typical days, two per monkey. Recall that performance improves as FPR decreases and TPR increases. Hence, while most differences in performance are not statistically significant, SWLDA results in better performance in 7 of the 12 cases (with lower FPRs and either higher TPRs or at least TPRs within $1\%$ of the best TPR). Furthermore, FPRs achieved by SWLDA were always the lowest, even in cases when the TPR achieved by another method was higher. Given its good performance and ease of implementation, SWLDA was selected for further analysis and for online error-classification.

**Table 1.** Comparison of error detection with different classifiers during initial brain-control. True positive rate (TPR) and false positive rate (FPR) estimated using five-fold cross validation from data recorded during initial brain control. Thresholds were selected to limit FPR below $5\%$, or to obtain the minimum positive FPR if it remained above $5\%$ for all the evaluated thresholds. MRS, middle-ring-small; SWLDA, step-wise linear discriminate analysis; QDA, quadratic discriminate analysis; SVM, Support vector machine. Best performance for each combination of monkey, day, finger group and direction of movement is marked in Bold along with the corresponding classifier.

			Monkey N				Monkey W			
			day 1		day 2		day 1		day 2	
			FPR	TPR	FPR	TPR	FPR	TPR	FPR	TPR
Index		SVM	5.4	62.4	5.3	68.9	5.1	42.6	5.3	32.7
	Extension	QDA	5.4	48.8	5.5	53.6	5.1	44.4	5.3	**52.2**
		**SWLDA**	**4.1**	**63.8**	**4.2**	**78.0**	**2.8**	**44.8**	**3.5**	46.9
		SVM	5.2	**62.9**	5.0	53.7	8.8	77.4	5.4	45.8
	Flexion	QDA	8.2	46.1	5.0	28.8	8.8	**84.5**	5.4	51.8
		**SWLDA**	**4.3**	62.3	**4.0**	**54.5**	**3.5**	58.9	**3.2**	**63.6**
MRS		SVM	5.4	53.8	5.3	56.8				
	Extension	QDA	8.3	43.6	5.5	40.9				
		**SWLDA**	**4.2**	**56.0**	**4.7**	**47.9**				
		**SVM**	5.2	**48.1**	5.0	**55.4**				
	Flexion	QDA	5.5	30.4	5.0	36.2				
		**SWLDA**	**4.3**	35.8	**3.9**	37.5				

Operational classifiers, i.e. those used online, were trained on 70% of a balanced set of labeled segments from initial brain control, and the threshold was selected from the ROC computed from the remaining 30%. During online operation, the decoded movement of each finger group was monitored to detect overlapping segments of *N* = 4 bins in which the finger-group was consistently flexing or extending. The corresponding neural data (from the *N* = 4 bins) was sent to the corresponding error-classifier for the relevant finger-group and movement type. Finally, the output of the classifier determined whether the movement was erroneous or not.

Further analysis was conducted to investigate how error-detection is affected by the location of the virtual fingers, either outside-the-target or inside-the-target. To assure enough training samples, this analysis was conducted on labeled segments from both initial brain control and the two phases of brain control with error monitoring. Three sets of classifiers were trained on balanced sets of: (a) all the labeled segments, (b) labeled segments outside-the-target, and (c) labeled segments inside-the-target. For fair comparison, the same number of training samples were used in each case (limited by the small number of labeled segments outside-the-target). The performance of the resulting classifiers was evaluated using five-fold cross-validation.

### 2.5. Error correction strategy

**Figure 5.**
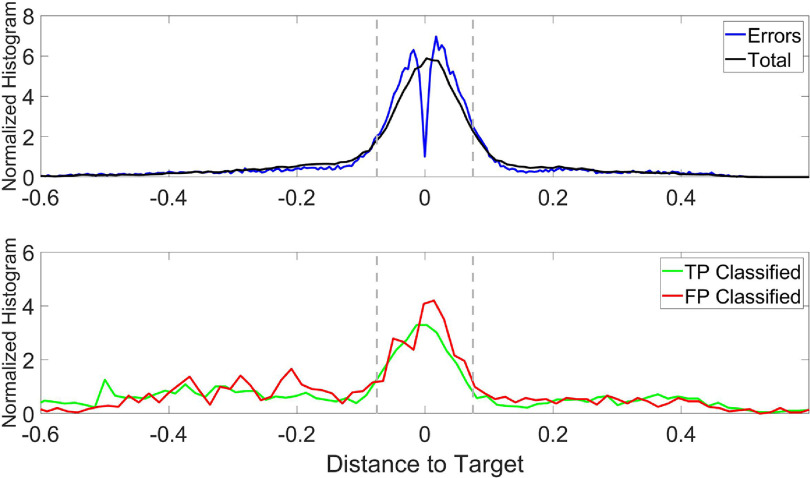
Normalized histograms of the distance of the MRS finger-group to its target (arbitrary units) during six sessions of brain control with error monitoring (without correction) performed over three days with Monkey N (days 1–3). When the distance is close to zero, the movements are usually not consistently away or toward the target, so there is a dip in the histogram of erroneous movements. Upper panel: distance during all movements (total, black) and during erroneous movements (Errors, blue). Lower panel: distance during TPs (green) and FPs (red). Grey dashed lines represent the range of the target.

In order to design an error-correction strategy, we investigated the distributions of the distance of the decoded finger position to the target, under four conditions: (1) distances at each time sample (total), (2) distances when erroneous movements occurred (errors), (3) distances when erroneous movements were correctly detected as erroneous (TPs), and (4) distances when correct movements were incorrectly classified as erroneous (FPs). Figure 5 presents the normalized histograms of the distance of the MRS finger-group to its target (in arbitrary units) during six sessions of brain control with error monitoring (without correction) performed over three days with Monkey N (Days 1–3). Dashed lines mark the target boundaries.

The means and standard deviations of the distances are $0.0013 \pm 0.19$ for all movements (total) and $ -0.0067 \pm 0.15$ when erroneous movements occurred. The standard deviations are relatively large, and in particular larger than the size of the target, due to the long tails of the distributions. Nevertheless, $68\%$ of the total distances and $64\%$ of the distances when erroneous movements occurred were within the boundaries of the target. Thus, we opted to correct erroneous movements with a stopping strategy. Stopping is a good compromise between slowing and reversing erroneous movements. To assure meaningful correction and avoid oscillations and deadlock, we devised the following two-step stopping strategy: (a) movement was stopped for *N* bins (4–5 bins, 200–250 ms), (b) correction was paused for the next 2*N* bins (8–10 bins, 400–500 ms).

### 2.6. Error correction implementation and evaluation

Online correction was applied to the current output of the KF whenever an error detector detected an erroneous movement. The correction was implemented within the Python environment and the corrected kinematics was sent back to both the KF for the next update and to the xPC to control the virtual fingers. Error classifications were also sent to the xPC and logged there for offline analysis.

Various metrics are used to assess online performance of BMIs. In this study, we focus on two metrics: (a) orbiting time (OT), and (b) total time to target (TTT). As illustrated in figure 2, OT is the duration between the time a finger-group first enters the target (time to first acquire, TFA), and the time after which it remains in target until the end of trial. OT depends on the finger-group, and can be zero if the finger-group never leaves the target after TFA until the end of trial. TTT is the total time of the trial minus the hold time. Since trials ended only after both finger-groups remained within the target range for the hold duration, TTT is independent of the finger-group. These metrics assess the speed and accuracy of online task performance rather than the accuracy of error classification, which was assessed by the ROC.

The hypotheses that TTT and OT are shorter with error correction than without error correction were tested using one-sided Wilcoxon rank-sum test. The analysis was performed by comparing all trials from the first phase of brain control with error correction (B, see section 2.2) with all trials from the preceding and proceeding phases of brain control with error monitoring only (A, see section 2.2).

## 3. Results

### 3.1. Offline analyses

Offline analysis was conducted on neural activity (binned SBP) recorded during finger control and initial brain control performed by Monkey W (single-finger task, two different days) and Monkey N (two-finger task, six different days).

#### 3.1.1. Variance analysis

**Figure 6.**
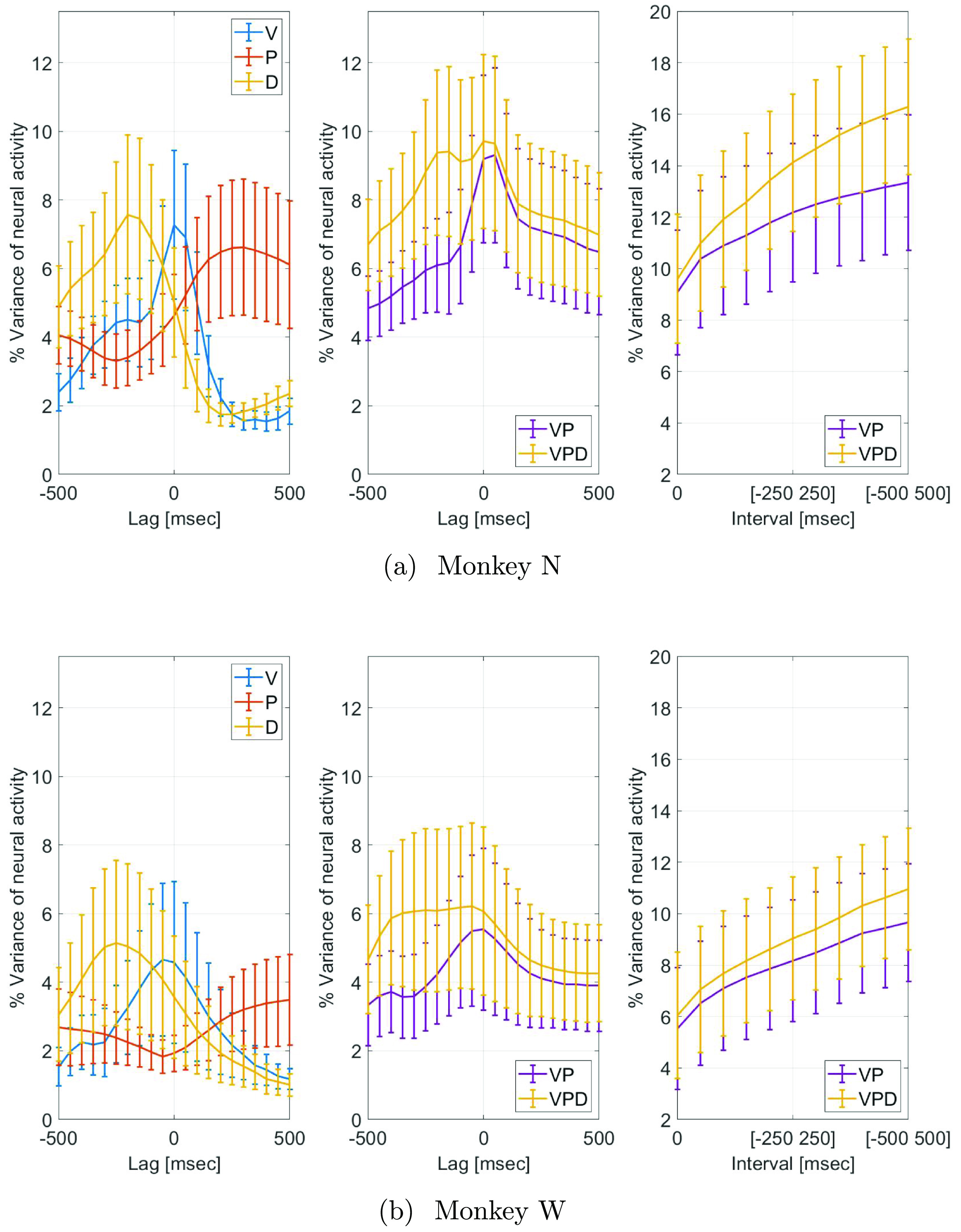
Percent variance of neural activity explained by velocity (V) position (P) distance to target (D), and their combinations (VP and VPD) during finger control performed by Monkey N on day 1 (a) and Monkey W on day 2 (b). Left and middle panels depict the mean percent variance explained by a single-lag model (equation (1)) as a function of the relative lag between the neural activity and the kinematics, where negative lags correspond to neural activity that occurs after the kinematics. Right panels depict the mean percent variance explained by a multi-lag model (equation (2)), as a function of the size of the interval of lags. Bars indicate standard error of the mean.

Figure 6 presents typical graphs of percent variance of neural activity that is explained by different kinematic variables (position, P, velocity, V and distance to target D) and their combinations (VP and VPD), during finger control performed by Monkey N (figure 6(a)) and Monkey W (figure 6(b)). The left and middle panels depict the percent variance of neural activity that is explained by a single-lag model (equation (1)) as a function of the relative lag, where negative lags correspond to neural activity that occurs after the kinematics. The percent variance of the neural activity that is explained by the velocity peaks at 0 ms or −50 ms for Monkey N and Monkey W, respectively, while the percent variance explained by the distance peaks when the neural activity lags the distance by −200 ms or −250 ms, respectively. Finally, the percent variance explained by the position peaks when the neural activity leads the position by 300 ms for Monkey N and by over 500 ms for Monkey W.

The lag between the neural activity and the distance agrees with the interpretation that the neural activity is related to processing the observed distance to the target. In any case, the peak of the variance of the neural activity that is explained by the distance cannot be attributed exclusively to the variance explained by the velocity since the peak correlation between the distance and the velocity is only 0.71 for monkey N and 0.75 for Monkey W and the correlation between the distance and the position is negligible (correlations are not shown). The hypothesis that the neural activity is related to the distance independently of the velocity is further supported by the right panels of figure 6, which depict the percent variance of the neural activity explained by a multi-lag model (equation (2)) as a function of the interval of lags. Focusing on the interval that ends at the lag at which the percent variance explained by the distance peaks (i.e. $(-200,200)$ ms for Monkey N and at $(-250,250)$ ms for Monkey W), VPD is significantly larger than the percent variance explained by VP (*p* < 0.05), one-sided Wilcoxon test). The same behavior was observed in all the days that were analyzed (six days with Monkey N and two days with Monkey W). Additional significance analysis, comparing the percent variance explained by P, V and D, is detailed in appendix A.

While we focus mainly on encoding of the distance to the target, we also note that the lag between the neural activity and position may be attributed to encoding of the desired or expected position. In any case, the peak of the variance of the neural activity that is explained by the position cannot be attributed exclusively to the variance explained by the velocity since the cross-correlation between the position and velocity is small (below 0.1 in magnitude) and does not exhibit a peak (correlations not shown).

Thus, the neural activity encodes not only the position and velocity but also the distance to the target. This suggests that changes in neural activity across multiple bins includes information about whether the finger moves away or toward the target, as the distance to the target would increase or decrease, respectively.

#### 3.1.2. Channel contribution

**Figure 7.**
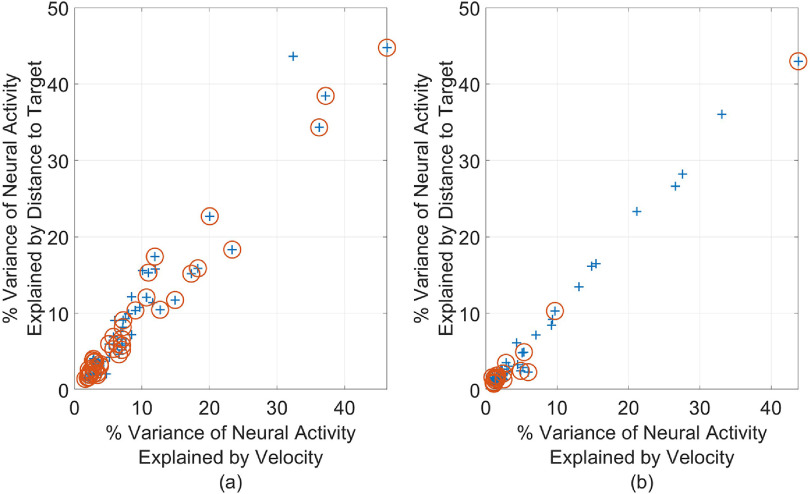
Maximum percent variance of neural activity at a single history bin explained by either the distance to the target (*y*-axis) or the velocity (*x*-axis) for each channel during finger control. Only channels used by the Kalman filter are shown. The channels marked by orange circle are also used by the SWLDA error classifier. (a) Monkey N, day 1, (b) Monkey W, day 2.

Additional analysis was conducted to investigate how different channels contribute to velocity and distance encoding in those days, and which channels were picked for error-detection. For that purpose, the maximum percent variance of neural activity that can be explained by each kinematic variable at a single lag was determined. Figure 7 depicts the maximum percent variance explained by the distance to target as a function of the maximum percent variance explained by the velocity for each channel that was used by the KF, for Monkey N (left panel) and Monkey W (right panel). Interestingly, the relationship is close to linear ($R^2 = 0.94$ and $R^2 = 0.98$ for Monkey N and Monkey W, respectively), suggesting that channels that actively encode the velocity are also active in encoding the distance to target.

Channels for which at least one bin of their activity was picked by the SWLDA to detect erroneous movements are marked by red circles ($66.7\%$ and $31.2\%$ of the channels used for the KF were picked by the SWLDA for Monkey N and Monkey W, respectively). Interestingly, the channel with the highest percent of variance that is explained by distance was picked by the SWLDA for both monkeys. However, at least for Monkey W, other channels for which the distance explains a large percent of the variance of the neural activity were excluded. In contrast, many of the channels picked by the SWLDA classifiers were those for which only a small percent of variance was explained by the distance.

To better understand these phenomena, we trained two additional sets of error-detectors based on the data recorded during initial brain control performed by Monkey W. The first set was forced to use the eight channels with percent variance explained by distance larger than $10\%$. The resulting ROCs were very similar to those obtained with the standard SWLDA, with AUC within $2\%$ of each other. This indicates that those channels do not encode additional information about erroneous movements.

The second set of error-detectors was trained without the channels for which the distance explains less than $2\%$. The resulting ROCs were worse than those obtained with the standard SWLDA with above $25\%$ reduction in the AUCs. A possible explanation might be that even the small percent of variance explained by distance by those channels was important since it was not correlated with the activity of the other channels picked by the SWLDA. Furthermore, those channels may encode other signals involved in error processing, including the deviation between the actual and expected position or control signals required to correct the movement.

#### 3.1.3. Classifier performance

**Figure 8.**
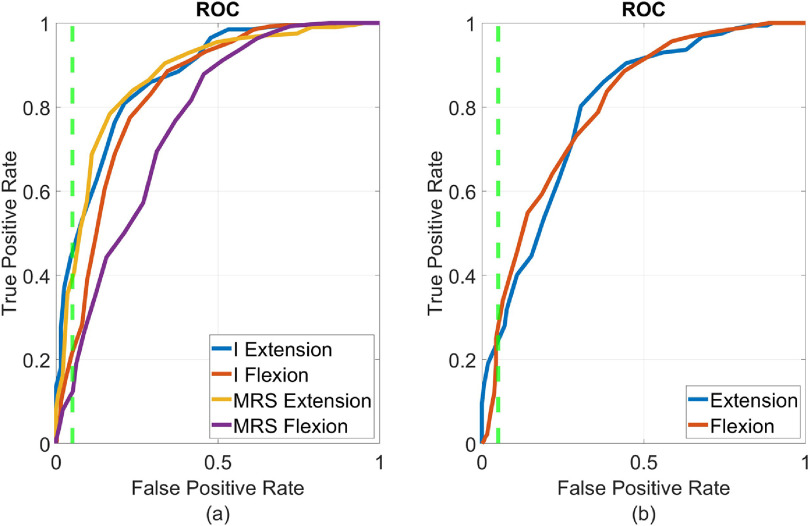
Receiver operating curves (ROCs) of the operational classifiers for each finger-group and movement type (flexion and extension) computed from a balanced set of labeled validation segments recorded during initial brain control. ROC describes the trade-off between true positive rate (TPR, i.e. the rate at which segments away from the target are correctly classified as such) versus false positive rate (FPR, i.e. the rate at which segments toward the target are mistakenly classified as away from the target) as the threshold is varied. Vertical dashed lines indicate the FPR$ = 5\%$ limit. (a) Monkey N, day 1. (b) Monkey W, day 2.

Offline performance of the operational error-classifiers was quantified by the ROC (see section 2.4). Typical ROCs, evaluated on validation data collected during initial brain control performed by Monkey N and Monkey W, are depicted in figure 8. The limit of FPR equal to $5\%$ is marked by a dashed vertical line. The best TPRs for FPR below $5\%$ are listed in the corresponding lines in table 2 (day 1 for Monkey N and day 2 for Monkey W), along with the AUCs. Results from five additional days with Monkey N and an additional day with Monkey W, are also listed. While FPRs are close to $5\%$ (ranging from $2.1\%$ to $4.9\%$) by design, TPRs range from $7.9\%$ to $64.7\%$ depending on the day, finger-group and movement type, with mean $26.9\%$ (mean of $28.2\%$ for the 6 days with Monkey N and $19.2\%$ for the two days with Monkey W).

**Table 2.** Offline performance evaluated from data recorded during initial brain control. ‘M’ and ‘D’ denote the Monkey and day. ‘T’ and ‘F’ denote TPRs (true positive rate) and FPRs (false positive rate) evaluated from ROCs computed from validation data. ‘A’ denotes AUC (area under the receiver operating curve). MRS represents the middle-ring-small finger group. FC denotes the number of firing channels with an average firing rate over 1 Hz.

		Flexion						Extension						
		Index			MRS			Index			MRS			
M	D	F	T	A	F	T	A	F	T	A	F	T	A	FC
N	1	4.9	21.5	0.83	2.1	8	0.76	4.5	44.2	0.87	3.5	35.7	0.87	26
	2	3.5	23.7	0.85	3.7	15.5	0.81	3.9	58.3	0.93	3	26.2	0.84	35
	3	3.9	51.6	0.91	4.6	19.4	0.74	4.3	34.9	0.84	3.5	17.2	0.82	20
	4	3.4	14.2	0.73	3.1	14.2	0.81	2.2	64.7	0.95	3.7	10.3	0.76	41
	5	4	24.1	0.80	4.6	16.1	0.75	4	24.2	0.81	4	33	0.84	47
	6	4.5	35.1	0.86	3.6	19.5	0.79	4.6	34.9	0.86	4	29.5	0.80	32
W	1	3.6	18.2	0.75				2.2	7.9	0.61				19
	2	4.3	25	0.80				3.8	25.5	0.80				11

The results reported in table 2 indicate that the variability in TPRs is associated with the variability in AUCs, which range from 0.61 to 0.95. Specifically, TPRs increase with AUCs with $R^2 = 0.73$ when considering the data from both monkeys, and $R^2 = 0.81$ when considering only the data from Monkey N. This implies that the overall quality of the detection varied, and not only the highest TPRs for FPRs below $5\%$. This variability cannot be attributed to the variability in the number of firing channels (channels with average firing rate above 1 Hz, see section 2.1), reported in the last column of table 2 ($R^2 = 0.02$ for data from both monkeys and $R^2 = -0.12$ for data from Monkey N only). In particular, the number of firing channels in the 4th day is second largest, and indeed on that day the TPR for index extension is the largest, but the TPR for error-detection during flexion is poor. We also did not see any correlation with the sample size (the number of erroneous segments during initial brain control, which were used for training and validation). Instead, the variability in AUCs may be attributed to the information available in the recorded channels. Specifically, error-detection would depend on the availability of channels that are highly tuned to the distance to the target, or to other signals associated with error processing, for the specific finger group and type of movement.

### 3.2. Real-time error detection

Brain control with error monitoring and error correction was performed by Monkey N during two days (day 1 and 2), using the error-detectors and thresholds that were determined using data from initial brain control. The resulting online performance, summarized in table 3, indicates that FPRs ranged from $2\%$ to $8.1\%$ with mean of $4.7\%$, while TPRs ranged from $5.7\%$ to $57.1\%$ with a mean of $28.1\%$. Comparing to offline performance (for the same two days), mean online FPR was $1\%$ higher than mean offline FPR, but remained below the $5\%$ threshold. Mean online TPR was $1\%$ lower than mean offline TPR for the same days. Thus, there was only a small degradation in performance (recall that performance improves as FPR decreases and TPR increases). It is important to note that offline performance was measured from relatively small data-sets ($30\%$ of segments from initial brain control).

**Table 3.** Online performance evaluated during brain control with error monitoring and brain control with error correction. TPRs (true positive rate) and FPRs (false positive rate) obtained online. MRS represents the middle-ring-small finger group.

		Flexion				Extension			
		Index		MRS		Index		MRS	
Monkey	Day	FPR	TPR	FPR	TPR	FPR	TPR	FPR	TPR
	1	2	13.1	2.4	5.6	8.1	40.2	7.4	38.7
N	2	2.7	16.8	5.7	23.7	4.1	57.1	4.9	29.8

**Figure 9.**
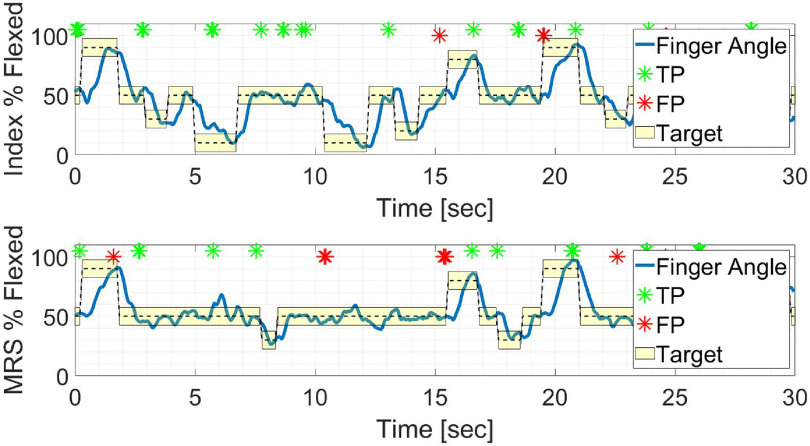
Examples of real-time error detection during a 30 sec section of brain control with error detection performed by Monkey N on day 1. Decoded finger positions (blue line) along with target locations (shaded yellow area, centered around the dashed black line). TPs, i.e. correct classification of movements away from the target’s center as erroneous, are marked with green stars on the top and FPs, i.e. incorrect classification of movements toward the target’s center as erroneous are marked with red stars on the top.

Figure 9 demonstrates a typical section of 30 s of brain control with error monitoring performed by Monkey N (day 1). The position of the Index finger and its target are shown in the top panel, while the position of the MRS finger-group and its target are shown in the bottom panel. TPs and FPs are marked by green and red stars, respectively. Cases of correct error detection (TPs) occurred, for example, when the index finger moved within the target between 7 and 10 section A case of correct error detection occurred when the MRS finger-group moved outside the target around 6 section. Finally, cases of incorrect error detection (FPs) occurred when the target of the index-finger switched around 19 s and 24 section If these FPs are corrected, it could degrade performance, and thus these are the instances we desire to minimize by restricting FPR to below $5\%$.

### 3.3. Online error-correction

**Figure 10.**
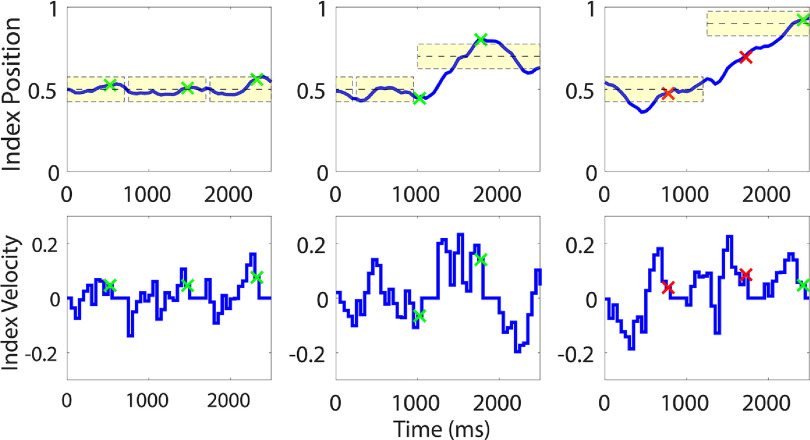
Selected examples of kinematic effects of online correction with Monkey N on day 1. Top panels: Corrected finger positions (blue line) along with target locations (shaded yellow area, centered around the dashed black line). Bottom panels: Corrected velocity of the index finger. Corrections in response to TPs are marked by green stars, while corrections in response to FPs are marked by red stars.

Movements that were detected as erroneous were corrected using the stopping strategy detailed in section 2.6. Specifically, the velocity of the corresponding finger group was set to zero for *N* = 4 bins, and correction was paused for the next $2N = 8$ bins. Representative sections of position and velocity of the index-finger during brain control with error correction are presented in figure 10. The time points when errors were detected are marked by green x’s for TPs and red x’s for FPs. Note that after each error-detection the velocity is zero for four bins. The left panels depict three instances of correct error-detection in which error correction kept the finger within the target long enough to satisfy the hold condition for both finger-groups. The middle panels demonstrate two instances of correct error detection: correcting the first one prevented moving in the wrong direction, while correcting the second one reduced the overshoot. Finally, the right panels depict two instances of incorrect error-detection (followed by one instance of correct error-detection). The first error-correction, though made by mistake, did not affect performance (and even facilitated staying in the target). However, the second error-correction slowed the movement toward the target and increased the time to acquire the target. Nevertheless, correcting these FPs did not severely affect performance compared to other correction strategies such as velocity reversal, which would have driven the finger further away from the target.

**Figure 11.**
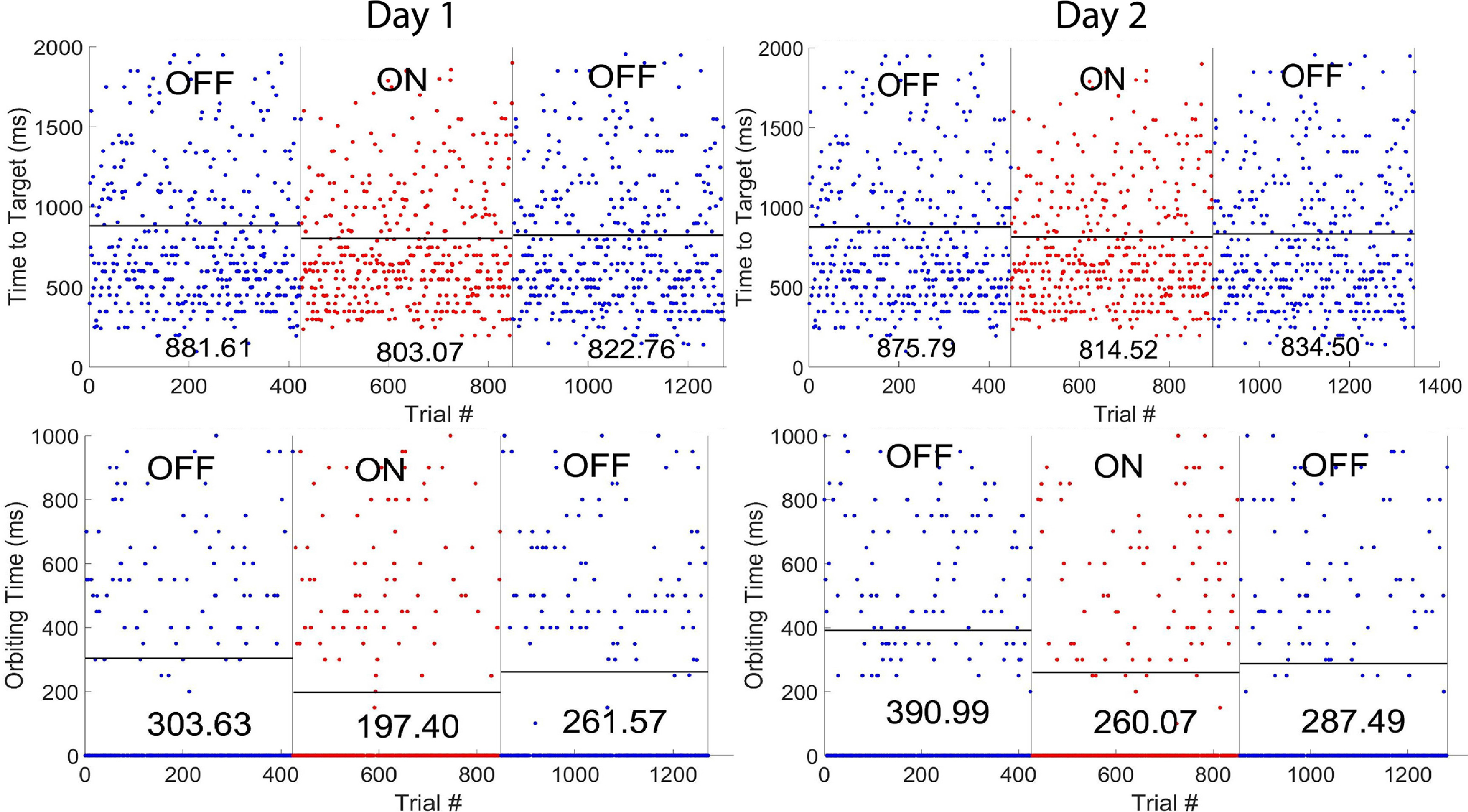
Effects of online error correction. Performance metrics evaluated during brain control with error monitoring (blue, OFF) and with error correction (red, ON) on two different days (day 1 and 2) performed by Monkey N. In each day, brain control with error detection was preceded and proceeded by brain control with error monitoring only, in an ABA fashion. Top panels: total time to target, TTT, which is the same for both finger-groups. Bottom panels: orbiting time (OT) for the MRS finger-group. Mean values for each phase are indicated and are marked by black lines. Both TTT and MRS’ OT were shorter when error correction was turned on, though only the effect on MRS’ OT was statistically significant.

The average effect of error correction on performance was assessed by evaluating the metrics described in section 2.6 during brain control with and without error-correction, and comparing between them. Figure 11 depicts the results from two experimental days with Monkey N, including TTT, which depends on both finger-groups, and OT for the MRS finger-group. Note that in many trials OT is zero. This occurred when the finger-group remained within the target once it entered the target until the end of the trial. Average TTT and OT went down after starting error correction (at the AB transition) by an average of 69.91 ms and 118 ms for TTT and OT respectively, and went up again after ending error-correction (after the BA transition) by an average of 19.84 ms and 45.8 ms for TTT and OT respectively.

To test the significance of these differences in performance, a one-sided Wilcoxon Rank-Sum test was performed comparing the OT within finger groups between the error monitoring and error correction trials. Mean OT for the MRS finger group was found to be significantly lower with correction on both days (p = 0.000 26, p = 0.0111) with reductions in OT of 30% and 23% respectively. This improvement in OT is consistent with the stopping error correction strategy, which facilitates remaining within the target.

### 3.4. Error detection inside and outside the target

Further analysis, detailed at the end of section 2.4, was conducted to evaluate the effect of the location of the virtual fingers, either outside or inside the target, on error-detection. This analysis was conducted on labeled segments from both initial brain control and the two phases of brain control with error monitoring (the two OFF sections analyzed in figure 11) recorded during experiments with Monkey N. For fair comparison, the same number of training segments were used for all classifiers. The analysis was performed using five-fold cross-validation and ROCs were computed from validation results in all folds.

**Figure 12.**
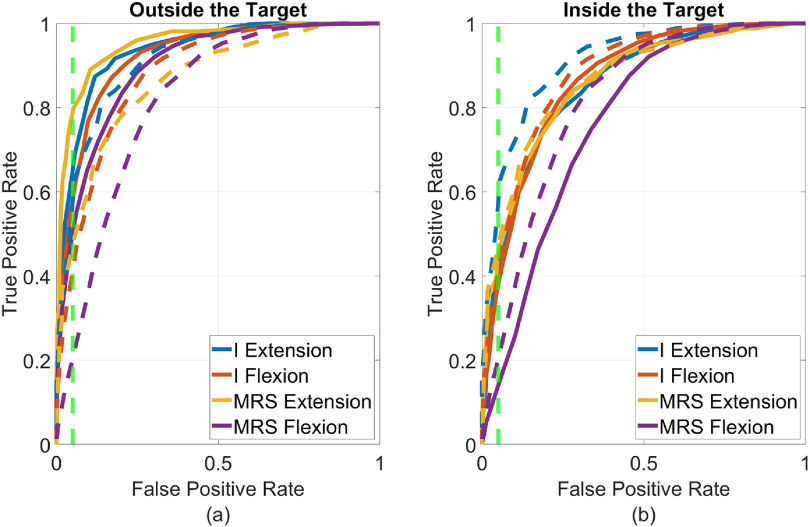
Effect of the location of the virtual fingers, either outside or inside the target, on error-detection. The receiver operating curves (ROCs) depict validation performance of different classifiers, trained and validated on balanced sets of labeled segments outside the target (a, solid lines) inside the target (b, solid lines) or both (dashed lines), using five-fold cross validation. The analysis was performed on data recorded from Monkey N (day 1) during initial brain control and during brain control with error monitoring, with the same number of training segments for all classifiers. Vertical dashed lines indicate the FPR$ = 5\%$ limit.

Figure 12 depicts the ROCs of error-classifiers trained and validated on movement segments recorded during day 1 with Monkey N outside the target (a, solid lines) or inside the target (b, solid lines) compared to ROCs for all movement segments, both inside and outside the target (dashed lines). It is evident that classifiers trained and validated on segments outside the target have better ROCs (larger TPRs for same FPRs) than classifiers trained and validated on all segments, or on segments inside the target.

Table 4 summarizes the average TPRs (for FPRs below 5%), over two days with Monkey N and over both finger groups and movement types. Different rows specify the location of training segments (all, outside or inside the target) and different columns specify the location of validation segments from which the ROCs were computed. Independent of the location of the training segments, TPRs are always highest when the ROCs are computed from validation segments outside the target. Furthermore, TPRs obtained by classifiers that were trained and validated on segments outside the target are significantly larger than TPRs obtained by classifiers that were trained and validated on segments inside the target or on segments from both inside and outside the target (one sided t-test, $p\lt10^{-14}$). Thus, detecting erroneous movements outside the target is more accurate than inside the target.

**Table 4.** Average AUC (area under the curve) and average maximum TPR (true positive rate) for FPR (false positive rate) below $5\%$ from ROCs (receiver operating curves) of error classifiers trained and evaluated on balanced sets of labeled segments from different locations with respect to the target (all includes both segments outside and inside the target). AUCs and TPRs are averaged over both days in which brain control with error monitoring was conducted with Monkey N (day 1 and 2), finger groups, and movement types. For fair comparison the same number of training segments were used for all classifiers.

		Validation					
		All		Outside the target		Inside the target	
		TPR	AUC	TPR	AUC	TPR	AUC
	All	37.1	0.86	51.7	0.89	31.3	0.83
Training	Outside the target	34.4	0.85	58.1	0.92	28.6	0.8
	Inside the target	35.7	0.85	40.3	0.85	33.8	0.84

## 4. Discussion

In this work we demonstrate that erroneous movements can be detected from neural activity recorded in the primary motor cortex, within a time frame that may enable them to be used to improve online BMI performance. The ability to detect erroneous movements was demonstrated offline on two monkeys, but only one monkey (Monkey N) was available for online error monitoring and error correction. Online experiments with Monkey N were replicated on two days to make sure that the monkey did not adopt an unusual strategy on the first day. Indeed, the reported improvement in performance was significant on both of those days.

First we show that including distance to target, rather than just kinematics, can explain significantly more variance in the neural activity recorded in the primary motor cortex, suggesting that neural activity in motor cortex not only encodes position and velocity but also distance to the target. The percent variance explained by the distance peaks when the neural activity lags the distance by about 200–250 ms (4–5 bins), while the percent variance explained by the velocity peaks at a lag of 0–50 ms. The lag between the neural activity and the distance agrees with the interpretation that the neural activity is partially related to processing the observed distance to the target. Thus, the neural activity encodes not only the position and velocity but also the distance to the target. This suggests that the neural activity across multiple bins include information about whether the finger moves away or toward the target, as the distance to the target would increase or decrease, respectively.

Motivated by these results and insights we used *N* = 4 bins of neural activity (200 ms), from all the units used for the BMI, to detect erroneous movements. Error detection was applied only to segments of *N* = 4 bins in which the movement type was consistent (either flexion or extension). We note that selecting a larger number of bins might have provided more information, but would have also reduced the number of segments with consistent movement type. Selecting thresholds that limit FPRs to below $5\%$, this strategy resulted in an average offline TPR of $28.2\%$ for Monkey N and $19.2\%$ for Monkey W. Using the same thresholds online resulted in an average online TPR of $28.1\%$ for Monkey N. Note that the average online FPR remained below $5\%$ even-though the thresholds were selected offline. However, in general, online FPRs cannot be guaranteed to remain below $5\%$. Nevertheless, in real-life human applications, both error detectors and KF decoders could be re-calibrated via a new training session with known targets if performance was observed to degrade by the user.

The demonstrated ability to detect erroneous movements might be related, as suggested above, to neural encoding of the distance to the target, but may also be related to direct error processing in the motor cortex. This is consistent with an fMRI study [16] that revealed that execution errors activated clusters in M1. It has also been shown that BMI outcome errors can be detected from neural activity in the motor cortex [4]. In that work, errors were detected at the end of the trial, when the cursor was close to the wrong target. Instead, here we demonstrate that it is possible to detect execution errors during continuous movements.

Interestingly, we demonstrate that error-detectors trained and applied to erroneous movements outside the target have better validation performance than error-classifiers trained and applied to erroneous movements inside the target (sub-section 3.4). This suggests that erroneous movements outside that target evoke more distinguished patterns of error processing.

To overcome erroneous movements we applied a simple stopping strategy: the movement was stopped for *N* = 4 bins and correction was paused for an additional $2N = 8$ bins. Despite an average online TPR of $28.1\%$ and average online FPR close to $5\%$ and despite requiring 200 ms to detect and react to a suspected error, we saw a significant improvement in task performance via reduced OT of the MRS finger-group. The significant reduction in MRS’ OT resulted in a reduction in TTT though it was not statistically significant. We note that other measures of performance that rely on the decoded velocity are less relevant for assessing online performance since the ground truth is unknown. In any case, additional study is needed to determine the best correction strategy, considering for example, slowing down, reversing the movement, or stopping and pausing for different number of bins.

The detection of erroneous movements from EEG activity evoked in response to errors, known as ErrPs, is more mature [6–11]. ErrPs are a special type of event related potentials (ERP), i.e. potentials that are time locked to discrete events. In the case of ErrPs, those events are the occurrence of errors. Thus, by nature, ErrPs detect discrete erroneous events, like selecting the wrong character [12, 13] moving in the wrong direction [14, 15], or reaching the wrong target. Instead, in this work we used continuous neural activity to detect erroneous movements away from the target.

Other groups have attempted to augment a continuous BMI decoder with a classifier running in parallel to determine when and how to modify the decoder’s output. For example Sachs *et al* showed that switching between fast and slow modes with different KF parameters significantly improves performance [32]. It has also been shown that explicitly decoding a stop state substantially reduces orbiting time, as shown in Kao *et al* [33]. Here we achieve a performance improvement with a different approach, detecting movements away from the target directly. While error-detection inside the target may function as a stop-recommender, the comparison analysis in sub-section 3.4 indicates that error detection performs especially well when restricted to erroneous movements outside the target. Thus, there may be a greater potential for improvement when considering both interpretations, since there is likely representation of both detected errors and ‘desired stopping’ within motor cortex.

Kao *et al* [33] demonstrated the power of decoding both analog and discrete state variables. Discrete states were decoded using a hidden Markov model. Depending on the Monkey, they distinguished between either move and stop states or slow, fast, and idle states. Thus, it is also interesting to investigate the ability to distinguish between in-target and outside-the-target states from the same neural activity used for the KF decoder. The resulting ROCs, depicted in appendix B, indicate that limiting the FPR to $5\%$, results in TPRs of 40%–57% for Monkey N (depending on the finger group and movement type) and around $30\%$ for Monkey W. Future work may evaluate how ‘in target’ detection improves error correction. In particular, the movement can be stopped when the ‘in target’ detector detects that the finger is inside the target, independent of the output of the error-detector. However, while the neural activity that enables error-detection is assumed to be related to moving away from the target, and hence to be independent of the characteristics of the target, the neural activity that enables ‘in target’ detection may depend on the characteristics of the target. Thus, it is left for future research to assess how well ‘in target’ detection will generalize to situations with different targets.

We chose the motor cortex because of the location of existing implants in our NHP. However, better error related signals could be found in other brain areas such as the anterior cingulate cortex (ACC) and the basal ganglia [34]. While there are challenges with implanting areas such as ACC or basal ganglia, new electrode technologies may allow for this in the future. Furthermore, error detection might be useful not only for real time error correction but also to re-training the KF, as has been suggested and developed for EEG-based BMIs [35].

A long term goal for BMIs would be to engage the natural pathways in the brain for quickly adapting to motor errors. For example, the cerebellum is thought to provide a rapid prediction of the outcome and expected feedback from actively executing motor commands as shown by Therrien *et al* [36]. This is helpful in generating active motor corrections online [37]. With more electrodes recording from throughout the motor pathway in the brain, the amount of time required to detect an error may be much lower than the 200 ms used here. Bypassing the time required by the spinal cord and muscles to act, it is possible that one day BMIs could provide more seamless integration with assistive technology than is possible using control signals from muscles or movements directly.

## Figures and Tables

**Figure B1.**
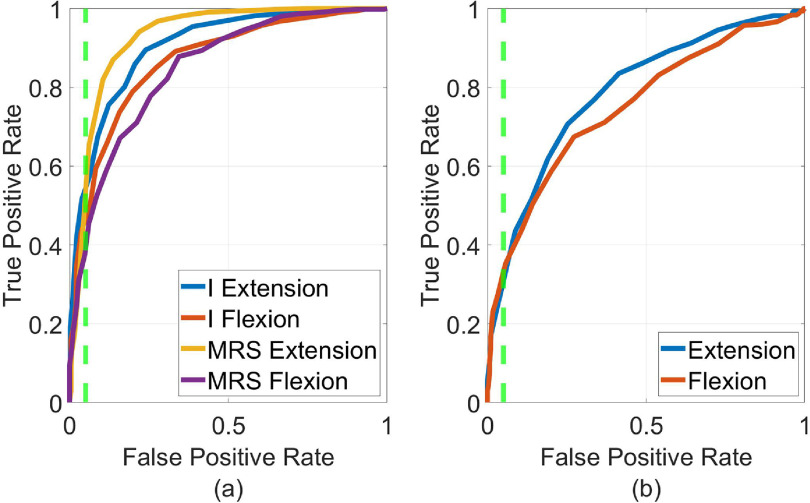
ROCs of ‘in target’ detectors trained on data recorded during initial brain control. TPR is the percent of cases when the fingers are inside the target and the detector correctly labels as inside, and FPRs are the percent of cases when the fingers are outside the target, and the detector mistakenly labels as inside. Vertical dashed lines mark the FPR = $5\%$ threshold. (a) Monkey N, day 1. (b) Monkey W, day 2.

## Data Availability

Data will be made available upon paper acceptance. The data that support the findings of this study will be openly available following an embargo at the following URL/DOI: https://chestekresearch.engin.umich.edu/data-and-resources/. Data will be available from 01 December 2023.

## Abbreviations

ACC—anterior cingulate cortex; EEG—Electroencephalogram; BMI—brain–machine interface; ERP—Event-related potential; ErrP—Error related potential; FP—False positive; FPR—False positive rate; KF—Kalman filter; M1—primary motor cortex; PCG—precentral gyrus; PMd—Premotor dorsal; MRS—middle-ring-small fingers; OT—Orbiting time; NHP—Non-human primate; QDA—Quadratic discriminate analysis; ROC—receiver operating curve; SBP—Spike-band power; SVM—Support vector machine; SWLDA—Step-wise linear discriminate analysis; TP—True positive; TPR—True positive rate; TTT—Total time to target; VP—Velocity and position; VPD—velocity, position and distance to target.

## Appendix A Comparison of percent variance explained by different variables

For completeness, we also compared the percent variance explained by the different kinematic variables at three intervals of $[-50,50]$ ms around the time at which the percent variance explained by each individual variable peaks (left panel of figure 6), table A1 summarizes the p-value of one-tailed Wilcoxon rank-sum test of the null hypothesis that the variance explained by the variable in the first column (X1) is smaller or equal to the variance explained by the variable in the second column (X2) at the indicated interval. Since there are 12 comparisons (3 for each monkey), a conservative threshold for significance, after Bonferroni correction, is 0.004. The results indicate that the percent variance explained by the distance around its peak is significantly larger than the percent variance explained by the other variables. This also holds for the percent variance explained by the position, but not for the percent variance explained by the velocity. In particular, the percent variance explained by the velocity, around its peak at 0 ms, is not significantly larger than that explained by distance, at least for monkey W.

**Table A1.** Significance analysis comparing the variance explained by different pairs of kinematics variables at different intervals. Wilcoxon rank-sum test was used to evaluate the null hypothesis that the variance explained by the variable in the first column (X1) is smaller or equal to the variance explained by the variable in the second column (X2) at the indicated interval. The tests were conducted using data recorded during finger control sessions in both days 1 and 2 performed by Monkey N or Monkey W.

Variance explained by X1 Vs. variance explained by X2 at indicated interval					
		Monkey N		Monkey W	
X1	X2	Interval	p-value	Interval	p-value
D	V	$[-250, -150]$	${\lt}0.0001$	$[-300, -200]$	${\lt}0.0001$
D	P	$[-250, -150]$	${\lt}0.0001$	$[-300, -200]$	0.00055
V	D	$[-50, 50]$	0.0007	$[-50, 50]$	0.085
V	P	$[-50, 50]$	0.022	$[-50, 50]$	${\lt}0.0001$
P	D	$[250, 350]$	${\lt}0.0001$	$[400, 500]$	${\lt}0.0001$
P	V	$[250, 350]$	${\lt}0.0001$	$[400, 500]$	${\lt}0.0001$

## Appendix B ‘In target’ detection

Motivated by Kao *et al* [33], we also evaluated the ability to detect when the virtual fingers are inside the target from the recorded neural activity used by the KF. Four detectors were trained depending on the finger-group and the type of movement (flexion or extension), based on data recorded during initial brain control. Positive responses indicate that the finger-group is inside the target. The resulting ROCs are depicted in figure B1. Restricting FPR to below $5\%$ results in TPRs between 40% and 57% for Monkey N and TPRs around $30\%$ for Monkey W.
